# Tris(1,10-phenanthroline)cadmium 3,3′-dicarb­oxy-4,4′-diazenedi­yl­dibenzoate–4,4′-diazenediyldiphthalic acid–methanol (1/0.5/1)

**DOI:** 10.1107/S160053681102068X

**Published:** 2011-06-11

**Authors:** Jun Wang, Lu Lu, Wu Wei-Ping, Xi-Yang He, Wang Tao

**Affiliations:** a Institute of Functionalized Materials, Sichuan University of Science and Engineering, Zigong 643000, People’s Republic of China; b College of Chemistry and Pharmaceutical Engineering, Sichuan University of Science and Engineering, Zigong 643000, People’s Republic of China

## Abstract

In the title compoud, [Cd(C_12_H_8_N_2_)_3_](C_16_H_8_N_2_O_8_)·0.5C_16_H_10_N_2_O_8_·CH_3_OH, the Cd^II^ atom has a distorted octa­hedral coordination formed by six N atoms from three separate phenanthroline ligands. One of the 4,4′-diazenediyldiphthalic acid mol­ecules is arranged around an inversion center and possesses two –COOH groups, while the other is partially deprotonated and is a dianion for charge balance. It can be noted that, in the undeprotonated acid, the –COOH groups are disordered over two positions by rotation around the C—C bond linking the –COOH group to the phenyl ring. Surprisingly, the H atom is not involved in the disorder. In the dianion, the remaining H atom is located between the two COO groups. These deprotonated and undeprotonated mol­ecules are linked by O—H⋯O hydrogen bonds, forming a chain developing parallel to the [111] direction. The methanol solvent molecule is highly disordered; it was  not considered in the final model by elimination of its contribution from the intensity data.

## Related literature

For background to crystal engineering, see: Yaghi *et al.* (2003 ▶); Kitagawa *et al.* (2004 ▶). For rigid carb­oxy­lic acids, see: Banerjee *et al.* (2008 ▶); Liu, Huang *et al.* (2011 ▶). For related chelating *N*-donor ligands, see: Liu, Jia & Wang (2011 ▶); Liu (2011 ▶); Breneman & Parker (1993 ▶). 
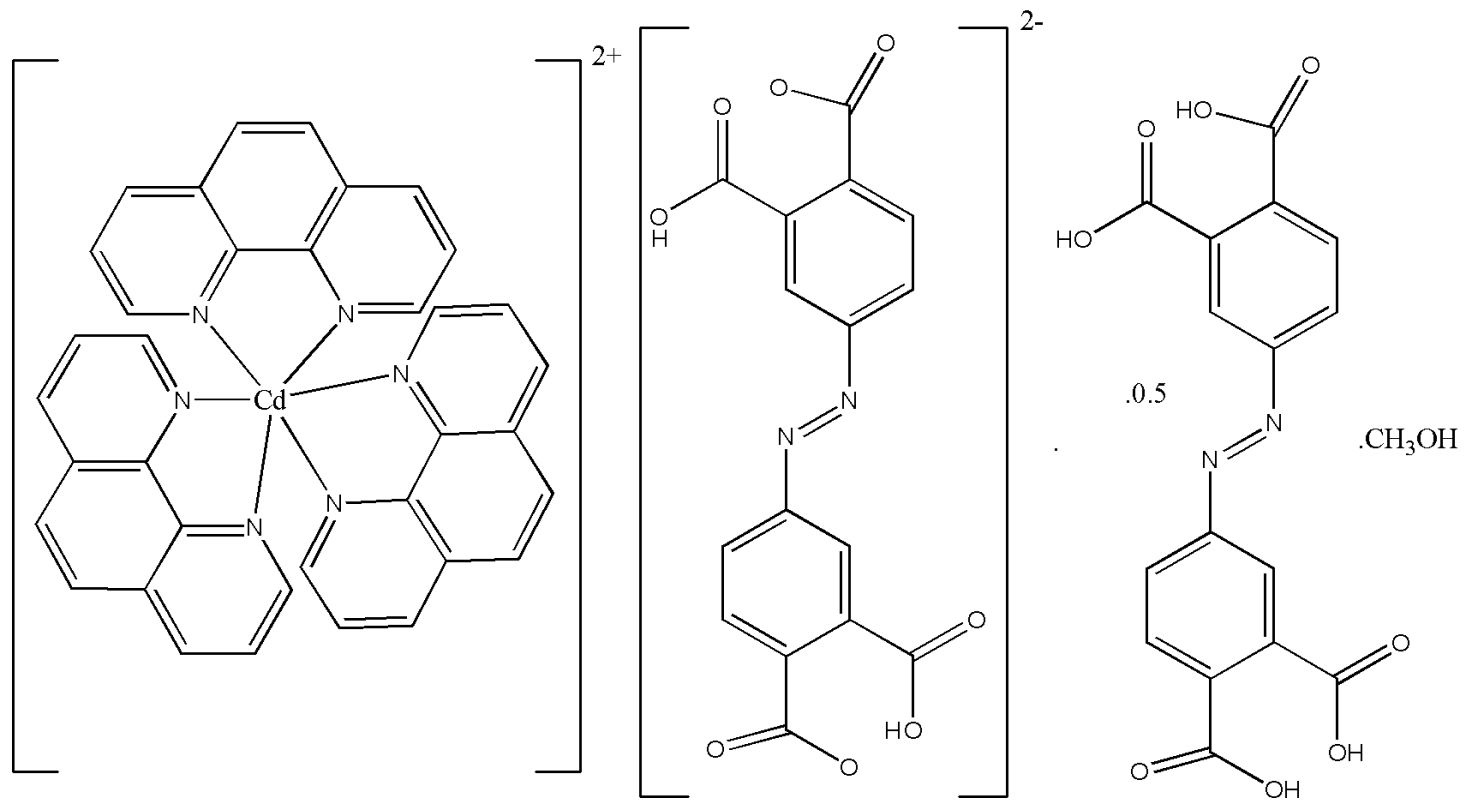


## Experimental

### 

#### Crystal data

[Cd(C_12_H_8_N_2_)_3_](C_16_H_8_N_2_O_8_)·0.5C_16_H_10_N_2_O_8_·CH_4_O
*M* *_r_* = 1220.43
Triclinic, 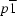

*a* = 13.6902 (9) Å
*b* = 13.7659 (9) Å
*c* = 16.9518 (11) Å
α = 79.022 (1)°
β = 73.492 (1)°
γ = 64.439 (1)°
*V* = 2754.7 (3) Å^3^
*Z* = 2
Mo *K*α radiation
μ = 0.47 mm^−1^
*T* = 298 K
0.23 × 0.16 × 0.07 mm

#### Data collection

Bruker APEXII area-detector diffractometer
Absorption correction: multi-scan (*SADABS*; Bruker, 2008 ▶) *T*_min_ = 0.900, *T*_max_ = 0.968
21086 measured reflections
10030 independent reflections
8170 reflections with *I* > 2σ(*I*)
*R*_int_ = 0.021

#### Refinement

*R*[*F* ^2^ > 2σ(*F* ^2^)] = 0.035
*wR*(*F* ^2^) = 0.103
*S* = 1.07
9876 reflections
775 parameters
10 restraints
H-atom parameters constrained
Δρ_max_ = 0.66 e Å^−3^
Δρ_min_ = −0.29 e Å^−3^

### 

Data collection: *APEX2* (Bruker, 2008 ▶); cell refinement: *SAINT* (Bruker, 2008 ▶); data reduction: *SAINT*; program(s) used to solve structure: *SHELXS97* (Sheldrick, 2008 ▶); program(s) used to refine structure: *SHELXL97* (Sheldrick, 2008 ▶) and *PLATON* (Spek, 2009 ▶); molecular graphics: *ORTEPIII* (Burnett & Johnson, 1996 ▶), *ORTEP-3 for Windows* (Farrugia, 1997 ▶) and *Mercury* (Macrae *et al.*, 2006 ▶, 2010 ▶); software used to prepare material for publication: *publCIF* (Westrip, 2010 ▶).

## Supplementary Material

Crystal structure: contains datablock(s) I, global. DOI: 10.1107/S160053681102068X/dn2688sup1.cif

Structure factors: contains datablock(s) I. DOI: 10.1107/S160053681102068X/dn2688Isup2.hkl

Additional supplementary materials:  crystallographic information; 3D view; checkCIF report

## Figures and Tables

**Table 1:** Hydrogen-bond geometry (Å, °)

*D*—H⋯*A*	*D*—H	H⋯*A*	*D*⋯*A*	*D*—H⋯*A*
O6—H6*A*⋯O7	1.15	1.24	2.386 (4)	174
O10—H10*A*⋯O11	1.10	1.41	2.367 (4)	141

**Fig. 1.**
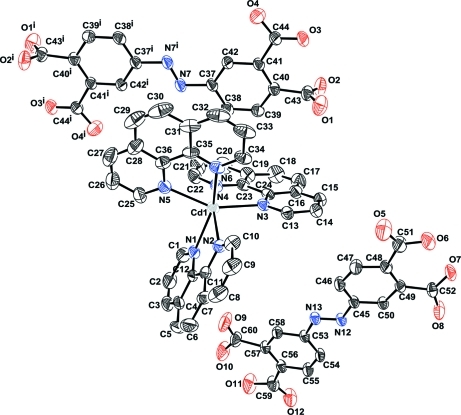
Molecular structure of (I), showing the atom-labelling scheme. Thermal displacement are drawn at the 30% probability level. Only the major components of the disordered carboxylate groups are represented. H atoms have been omitted for clarity. [symmetric codes: -x+1, -y, -z+1].

**Fig. 2.**
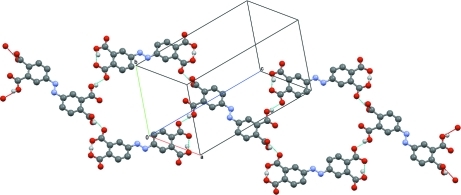
View of the 1D chain formed by the O-H···O hydrogen bonds linking the H4L and H2L molecules. Hydrogen bonds are shown as dashed lines. H atoms not involved in hydrogen bondings have been omitted for clarity.

## supplementary crystallographic information

### Comment

In the past decade, much progress has been achieved in the synthesis and structural characterization of metal-organic frameworks(MOFs) due to their potential applications (Yaghi *et al.*, 2003; Kitagawa *et al.*, 2004). Generally, the multidentate organic ligands containing coordination sites of O donors are widely used as building blocks in the construction of MOFs (Banerjee *et al.*, 2008; Liu, Huang *et al.*, 2011). On the other hand, 1, 10-Phenanthroline, one of those ligands, has usually been used to construct a great variety of structurally interesting entities, such as monomers(Breneman & Parker, 1993; Liu, Jia & Wang, 2011; Liu, 2011). Herein, we are interested in self-assemblies of Cd(II) ion with H_4_L and phenanthroline, which led to the preparation of the title compound.

In the asymmetric unit of title compound, there are one Cd(II) ion, three phen ligands, one deprotonated H_2_L, a half undeprotonated H_4_L ligand and one methanol molecule. As shown in Fig. 1. The Cd(II) atom is six-coordinated in a slightly distorted octahedral geometry defined by six N atoms from three different phen ligands. Interestingly, one of the (4,4'-diazenediyldiphthalic acid) is arranged around inversion center and possess two COOH groups, while the other is partially deprotonated and it is a dianion for balancing the charge. The Cd-N bond distances range from 2.329 (3) to 2.366 (3)Å. The N4-Cd1-N5 and N1-Cd1-N5 bond angles are 90.58 (9) and 93.19 (9)°, respectively. From the above values, it appears that the three phen ligands are nearly perpendicular to each other.

In H_2_L, the acidic H atom is nearly engaged in a bridging O···H···O interactions (Table 1). Furthermore, The molecules of H_4_L are linked by O-H···O hydrogen bonds to two H_2_L on both sides, forming a one-dimensional chain with void parallel to the [1 1 1] direction (Fig. 2, Table 1). The disordered methanol molecule is located in the void. The Cd(II) complexes are antiparallel to the above chains. The above hydrogen bonds could participate to the stabilization of the title complex.

### Experimental

The Cd(AC)_2_.H_2_O(19mg, 0.1mmol) was added dropwise slowly to ligand H_4_L(16mg, 0.06mmol) and phen (20mg, 0.01mmol) methanol solution(15mL). The pH of the mixture solution was adjusted to about 3.5 with 2N HAC solution. Then, the reaction mixture was stirred for 15 days at room temperature. Crystals of (I) were obtained at room temperature.

### Refinement

The occupancy of the COOH group was determined by fixing the sum of the occupancy to 1 and by using overall isotropic thermal parameter for O atoms and restraining the C-O distances by using the SAME instruction. The ratio was found to be equal to 0.65/0.35. Once the occupancy has been determined, the occupancy factors were fixed and the Uiso for the O atoms was refined freely then anisotropic thermal parameters were introduced.

All H atoms attached to C atoms were fixed geometrically and treated as riding with C—H = 0.93 Å, and U_iso_(H) = 1.2U_eq_(C).

All H atoms attached to the COOH groups were found in difference Fourier maps, and then they were refined freely with U_iso_(H) = 1.2U_eq_(C). In the last cycles of refinement they were treated as riding on their parent O atoms.

The unit cell contains a certain amount of methanol molecules. However, these molecules appear to be highly disordered and it was difficult to model their positions and distribution reliably. Therefore, the SQUEEZE function of PLATON (van der Sluis & Spek, 1990; Spek, 2003) was used to eliminate the contribution of the electron density in the solvent region from the intensity data, and the solvent-free model was emplyed from the final refinement.

There are two large cavities of about 113 \%A^3^ per unitl cell. PLATON estimated that each cavity contains 17 electrons which may correspond to a solvent molecule of methanol as suggested by chemical analyses.

### Crystal data

**Table:**

[Cd(C_12_H_8_N_2_)_3_](C_16_H_8_N_2_O_8_)·0.5C_16_H_10_N_2_O_8_·CH_4_O	*Z* = 2
*M**_r_* = 1220.43	*F*(000) = 1244
Triclinic, *p*1	*D*_x_ = 1.471 Mg m^−^^3^
Hall symbol: -P 1	Mo *K*α radiation, λ = 0.71073 Å
*a* = 13.6902 (9) Å	Cell parameters from 10030 reflections
*b* = 13.7659 (9) Å	θ = 2.4–25.2°
*c* = 16.9518 (11) Å	µ = 0.47 mm^−^^1^
α = 79.022 (1)°	*T* = 298 K
β = 73.492 (1)°	Block, red
γ = 64.439 (1)°	0.23 × 0.16 × 0.07 mm
*V* = 2754.7 (3) Å^3^	

### Data collection

**Table:**

Bruker APEXII area-detector diffractometer	10030 independent reflections
Radiation source: fine-focus sealed tube	8170 reflections with *I* > 2σ(*I*)
graphite	*R*_int_ = 0.021
φ and ω scans	θ_max_ = 25.4°, θ_min_ = 2.5°
Absorption correction: multi-scan (*SADABS*; Sheldrick, 2008)	*h* = −16→16
*T*_min_ = 0.900, *T*_max_ = 0.968	*k* = −16→16
21086 measured reflections	*l* = −20→20

### Refinement

**Table:**

Refinement on *F*^2^	Primary atom site location: structure-invariant direct methods
Least-squares matrix: full	Secondary atom site location: difference Fourier map
*R*[*F*^2^ > 2σ(*F*^2^)] = 0.035	Hydrogen site location: inferred from neighbouring sites
*wR*(*F*^2^) = 0.103	H-atom parameters constrained
*S* = 1.07	*w* = 1/[σ^2^(*F*_o_^2^) + (0.0643*P*)^2^] where *P* = (*F*_o_^2^ + 2*F*_c_^2^)/3
9876 reflections	(Δ/σ)_max_ = 0.002
775 parameters	Δρ_max_ = 0.66 e Å^−^^3^
10 restraints	Δρ_min_ = −0.29 e Å^−^^3^

### Special details

**Table:**

Geometry. All esds (except the esd in the dihedral angle between two l.s. planes) are estimated using the full covariance matrix. The cell esds are taken into account individually in the estimation of esds in distances, angles and torsion angles; correlations between esds in cell parameters are only used when they are defined by crystal symmetry. An approximate (isotropic) treatment of cell esds is used for estimating esds involving l.s. planes.
Refinement. Refinement of F^2^ against ALL reflections. The weighted R-factor wR and goodness of fit S are based on F^2^, conventional R-factors R are based on F, with F set to zero for negative F^2^. The threshold expression of F^2^ > 2sigma(F^2^) is used only for calculating R-factors(gt) etc. and is not relevant to the choice of reflections for refinement. R-factors based on F^2^ are statistically about twice as large as those based on F, and R- factors based on ALL data will be even larger.

### Fractional atomic coordinates and isotropic or equivalent isotropic displacement parameters (Å^2^)

**Table:**

	*x*	*y*	*z*	*U*_iso_*/*U*_eq_	Occ. (<1)
Cd1	0.194625 (14)	0.318919 (15)	0.770925 (10)	0.05192 (9)	
N1	0.2000 (2)	0.34134 (18)	0.90344 (14)	0.0644 (6)	
N2	0.01626 (18)	0.36033 (18)	0.85645 (13)	0.0572 (5)	
N3	0.15295 (17)	0.48840 (18)	0.69513 (12)	0.0523 (5)	
N4	0.36188 (17)	0.33952 (19)	0.70773 (13)	0.0564 (5)	
N5	0.29240 (19)	0.12915 (19)	0.78538 (14)	0.0620 (6)	
N6	0.16334 (18)	0.2430 (2)	0.67404 (13)	0.0606 (6)	
C1	0.2886 (3)	0.3350 (3)	0.9249 (2)	0.0823 (10)	
H1	0.3524	0.3293	0.8841	0.099*	
C2	0.2888 (4)	0.3367 (3)	1.0074 (3)	0.1024 (14)	
H2	0.3520	0.3315	1.0212	0.123*	
C3	0.1961 (5)	0.3460 (3)	1.0661 (2)	0.1059 (16)	
H3	0.1958	0.3469	1.1209	0.127*	
C4	0.0990 (4)	0.3544 (2)	1.04590 (18)	0.0860 (12)	
C5	−0.0037 (5)	0.3647 (3)	1.1045 (2)	0.1116 (18)	
H5	−0.0082	0.3654	1.1602	0.134*	
C6	−0.0918 (5)	0.3733 (3)	1.0815 (3)	0.1185 (18)	
H6	−0.1566	0.3798	1.1214	0.142*	
C7	−0.0899 (3)	0.3729 (2)	0.9969 (2)	0.0820 (10)	
C8	−0.1819 (3)	0.3822 (3)	0.9691 (3)	0.1015 (14)	
H8	−0.2486	0.3894	1.0067	0.122*	
C9	−0.1726 (3)	0.3806 (3)	0.8892 (3)	0.0946 (12)	
H9	−0.2326	0.3864	0.8704	0.114*	
C10	−0.0726 (2)	0.3703 (2)	0.8339 (2)	0.0712 (8)	
H10	−0.0679	0.3703	0.7781	0.085*	
C11	0.0094 (3)	0.3614 (2)	0.93751 (16)	0.0601 (7)	
C12	0.1051 (3)	0.3525 (2)	0.96180 (15)	0.0626 (8)	
C13	0.0526 (2)	0.5588 (2)	0.68553 (16)	0.0583 (7)	
H13	−0.0080	0.5412	0.7110	0.070*	
C14	0.0339 (3)	0.6570 (3)	0.63954 (18)	0.0697 (8)	
H14	−0.0374	0.7036	0.6338	0.084*	
C15	0.1219 (3)	0.6841 (3)	0.60295 (18)	0.0750 (9)	
H15	0.1111	0.7495	0.5713	0.090*	
C16	0.2285 (3)	0.6139 (2)	0.61277 (17)	0.0660 (7)	
C17	0.3245 (3)	0.6384 (3)	0.5782 (2)	0.0926 (11)	
H17	0.3167	0.7045	0.5485	0.111*	
C18	0.4240 (3)	0.5685 (4)	0.5879 (3)	0.0999 (12)	
H18	0.4841	0.5879	0.5663	0.120*	
C19	0.4426 (3)	0.4638 (3)	0.63071 (19)	0.0755 (9)	
C20	0.5488 (3)	0.3843 (4)	0.6386 (2)	0.0932 (12)	
H20	0.6116	0.3996	0.6170	0.112*	
C21	0.5585 (3)	0.2864 (4)	0.6775 (2)	0.0920 (11)	
H21	0.6279	0.2326	0.6811	0.110*	
C22	0.4630 (2)	0.2680 (3)	0.71172 (19)	0.0726 (8)	
H22	0.4705	0.2009	0.7394	0.087*	
C23	0.3510 (2)	0.4370 (2)	0.66705 (15)	0.0566 (6)	
C24	0.2412 (2)	0.5148 (2)	0.65882 (14)	0.0535 (6)	
C25	0.3575 (3)	0.0734 (3)	0.8377 (2)	0.0772 (9)	
H25	0.3637	0.1106	0.8752	0.093*	
C26	0.4165 (3)	−0.0373 (3)	0.8390 (3)	0.0953 (12)	
H26	0.4623	−0.0732	0.8758	0.114*	
C27	0.4066 (3)	−0.0921 (3)	0.7864 (3)	0.1054 (15)	
H27	0.4456	−0.1666	0.7869	0.126*	
C28	0.3383 (3)	−0.0383 (3)	0.7308 (2)	0.0828 (10)	
C29	0.3250 (4)	−0.0914 (4)	0.6713 (4)	0.1158 (17)	
H29	0.3618	−0.1660	0.6700	0.139*	
C30	0.2617 (4)	−0.0357 (4)	0.6188 (3)	0.1181 (18)	
H30	0.2533	−0.0728	0.5824	0.142*	
C31	0.2052 (3)	0.0796 (3)	0.6155 (2)	0.0846 (11)	
C32	0.1410 (3)	0.1409 (4)	0.5584 (2)	0.0984 (13)	
H32	0.1332	0.1070	0.5194	0.118*	
C33	0.0908 (3)	0.2482 (4)	0.5601 (2)	0.0996 (14)	
H33	0.0481	0.2894	0.5221	0.119*	
C34	0.1026 (3)	0.2983 (3)	0.61885 (17)	0.0736 (9)	
H34	0.0668	0.3730	0.6195	0.088*	
C35	0.2147 (2)	0.1351 (3)	0.67309 (17)	0.0629 (7)	
C36	0.2826 (2)	0.0746 (2)	0.73160 (18)	0.0635 (7)	
N7	0.48610 (19)	0.04145 (17)	0.51715 (13)	0.0576 (5)	
C37	0.4210 (2)	0.1386 (2)	0.47573 (14)	0.0520 (6)	
C38	0.3927 (2)	0.2337 (2)	0.50901 (16)	0.0608 (7)	
H38	0.4161	0.2321	0.5558	0.073*	
C39	0.3303 (2)	0.3303 (2)	0.47330 (16)	0.0599 (7)	
H39	0.3127	0.3939	0.4959	0.072*	
C40	0.2928 (2)	0.3353 (2)	0.40435 (15)	0.0526 (6)	
C43	0.2217 (3)	0.4439 (2)	0.3717 (2)	0.0631 (7)	
O1	0.1197 (5)	0.4801 (7)	0.3911 (5)	0.095 (2)	0.65
O2	0.2763 (8)	0.4987 (8)	0.3310 (6)	0.089 (3)	0.65
H2A	0.2330	0.5659	0.3341	0.107*	
O1B	0.1409 (11)	0.4604 (12)	0.3517 (11)	0.141 (8)	0.35
O2B	0.2661 (17)	0.5111 (14)	0.3622 (12)	0.099 (6)	0.35
C41	0.3216 (2)	0.2387 (2)	0.37008 (14)	0.0494 (6)	
C44	0.2883 (2)	0.2380 (2)	0.29324 (17)	0.0586 (7)	
O3	0.2267 (4)	0.3186 (3)	0.2611 (3)	0.0802 (12)	0.65
O4	0.3291 (4)	0.1439 (5)	0.2683 (4)	0.098 (2)	0.65
H4A	0.2887	0.1363	0.2425	0.117*	
O3B	0.3059 (11)	0.2912 (9)	0.2303 (5)	0.116 (4)	0.35
O4B	0.2695 (8)	0.1541 (8)	0.2944 (6)	0.083 (3)	0.35
C42	0.3845 (2)	0.1411 (2)	0.40667 (15)	0.0517 (6)	
H42	0.4023	0.0769	0.3848	0.062*	
N12	0.06170 (18)	1.02786 (18)	0.86767 (13)	0.0547 (5)	
N13	0.11991 (18)	0.94232 (17)	0.90151 (13)	0.0538 (5)	
O5	−0.0929 (4)	0.9112 (3)	0.5944 (2)	0.1661 (18)	
O6	−0.1637 (2)	1.0836 (2)	0.56001 (16)	0.1144 (10)	
H6A	−0.1735	1.1648	0.5767	0.172*	
O7	−0.18108 (17)	1.24773 (18)	0.60126 (13)	0.0793 (6)	
O8	−0.1673 (2)	1.30459 (18)	0.70774 (17)	0.0948 (8)	
O9	0.4026 (3)	0.6922 (2)	1.0424 (2)	0.1316 (12)	
O10	0.4367 (2)	0.7670 (2)	1.1261 (2)	0.1290 (12)	
H10A	0.4264	0.8509	1.1267	0.194*	
O11	0.3728 (3)	0.9370 (2)	1.1782 (2)	0.1315 (12)	
O12	0.2653 (3)	1.1037 (2)	1.15481 (17)	0.1148 (10)	
C45	0.0172 (2)	1.0163 (2)	0.80526 (15)	0.0519 (6)	
C46	0.0319 (3)	0.9203 (2)	0.78078 (19)	0.0736 (8)	
H46	0.0727	0.8546	0.8060	0.088*	
C47	−0.0152 (3)	0.9238 (3)	0.7178 (2)	0.0824 (10)	
H47	−0.0078	0.8591	0.7026	0.099*	
C48	−0.0734 (2)	1.0197 (2)	0.67622 (17)	0.0662 (8)	
C49	−0.0908 (2)	1.1174 (2)	0.70260 (15)	0.0514 (6)	
C50	−0.0443 (2)	1.1117 (2)	0.76747 (15)	0.0518 (6)	
H50	−0.0556	1.1760	0.7860	0.062*	
C51	−0.1117 (3)	1.0008 (3)	0.6058 (2)	0.0994 (12)	
C52	−0.1511 (2)	1.2309 (2)	0.66752 (18)	0.0610 (7)	
C53	0.1621 (2)	0.9608 (2)	0.96310 (15)	0.0488 (6)	
C54	0.1289 (2)	1.0597 (2)	0.99084 (17)	0.0595 (7)	
H54	0.0742	1.1203	0.9712	0.071*	
C55	0.1778 (3)	1.0679 (2)	1.04835 (18)	0.0651 (7)	
H55	0.1543	1.1352	1.0675	0.078*	
C56	0.2602 (2)	0.9811 (2)	1.07910 (16)	0.0571 (6)	
C57	0.2943 (2)	0.8789 (2)	1.05090 (16)	0.0546 (6)	
C58	0.2426 (2)	0.8717 (2)	0.99353 (15)	0.0532 (6)	
H58	0.2631	0.8046	0.9752	0.064*	
C59	0.3030 (3)	1.0095 (3)	1.1414 (2)	0.0810 (9)	
C60	0.3838 (3)	0.7717 (3)	1.0739 (2)	0.0827 (9)	

### Atomic displacement parameters (Å^2^)

**Table:**

	*U*^11^	*U*^22^	*U*^33^	*U*^12^	*U*^13^	*U*^23^
Cd1	0.05361 (13)	0.06166 (14)	0.04453 (12)	−0.02190 (9)	−0.01962 (8)	−0.00342 (8)
N1	0.0808 (16)	0.0558 (14)	0.0554 (13)	−0.0139 (12)	−0.0365 (12)	−0.0029 (10)
N2	0.0637 (14)	0.0519 (13)	0.0554 (12)	−0.0245 (11)	−0.0103 (10)	−0.0048 (10)
N3	0.0538 (12)	0.0627 (14)	0.0449 (11)	−0.0227 (11)	−0.0190 (9)	−0.0039 (10)
N4	0.0521 (12)	0.0708 (15)	0.0526 (12)	−0.0257 (11)	−0.0233 (10)	0.0007 (11)
N5	0.0610 (13)	0.0639 (15)	0.0584 (13)	−0.0244 (12)	−0.0127 (11)	−0.0012 (11)
N6	0.0547 (12)	0.0854 (18)	0.0498 (12)	−0.0323 (13)	−0.0129 (10)	−0.0117 (11)
C1	0.087 (2)	0.081 (2)	0.081 (2)	−0.0110 (18)	−0.0500 (18)	−0.0171 (17)
C2	0.135 (3)	0.078 (2)	0.100 (3)	−0.009 (2)	−0.084 (3)	−0.016 (2)
C3	0.192 (5)	0.053 (2)	0.065 (2)	−0.017 (2)	−0.069 (3)	−0.0049 (16)
C4	0.158 (3)	0.0394 (16)	0.0476 (16)	−0.0200 (19)	−0.038 (2)	−0.0007 (12)
C5	0.204 (5)	0.062 (2)	0.0389 (17)	−0.041 (3)	−0.003 (3)	−0.0063 (15)
C6	0.179 (5)	0.075 (3)	0.070 (3)	−0.053 (3)	0.027 (3)	−0.014 (2)
C7	0.111 (3)	0.0466 (17)	0.0665 (19)	−0.0316 (18)	0.0153 (18)	−0.0100 (14)
C8	0.088 (3)	0.076 (2)	0.126 (4)	−0.047 (2)	0.031 (2)	−0.027 (2)
C9	0.071 (2)	0.090 (3)	0.122 (3)	−0.042 (2)	0.007 (2)	−0.028 (2)
C10	0.0635 (18)	0.068 (2)	0.089 (2)	−0.0317 (15)	−0.0121 (16)	−0.0143 (16)
C11	0.0825 (19)	0.0346 (13)	0.0551 (15)	−0.0213 (13)	−0.0101 (14)	0.0010 (11)
C12	0.100 (2)	0.0324 (13)	0.0431 (14)	−0.0154 (14)	−0.0183 (14)	0.0010 (10)
C13	0.0577 (16)	0.0640 (18)	0.0541 (14)	−0.0205 (14)	−0.0224 (12)	−0.0019 (13)
C14	0.0742 (19)	0.068 (2)	0.0602 (17)	−0.0160 (16)	−0.0284 (15)	−0.0015 (14)
C15	0.097 (2)	0.0594 (19)	0.0598 (17)	−0.0251 (18)	−0.0210 (16)	0.0052 (14)
C16	0.078 (2)	0.0650 (19)	0.0563 (16)	−0.0317 (16)	−0.0158 (14)	0.0008 (13)
C17	0.100 (3)	0.084 (3)	0.094 (3)	−0.050 (2)	−0.015 (2)	0.014 (2)
C18	0.089 (3)	0.116 (3)	0.109 (3)	−0.066 (3)	−0.014 (2)	0.005 (2)
C19	0.0676 (19)	0.096 (2)	0.0718 (19)	−0.0449 (18)	−0.0179 (15)	0.0045 (17)
C20	0.061 (2)	0.136 (4)	0.095 (3)	−0.053 (2)	−0.0219 (18)	0.005 (2)
C21	0.0534 (18)	0.126 (3)	0.091 (2)	−0.033 (2)	−0.0298 (17)	0.018 (2)
C22	0.0569 (17)	0.090 (2)	0.0690 (18)	−0.0251 (16)	−0.0276 (14)	0.0075 (16)
C23	0.0578 (15)	0.0749 (19)	0.0478 (13)	−0.0322 (14)	−0.0185 (11)	−0.0052 (13)
C24	0.0620 (15)	0.0641 (17)	0.0421 (12)	−0.0283 (13)	−0.0173 (11)	−0.0054 (12)
C25	0.073 (2)	0.078 (2)	0.0716 (19)	−0.0264 (18)	−0.0208 (16)	0.0133 (16)
C26	0.074 (2)	0.078 (3)	0.105 (3)	−0.018 (2)	−0.014 (2)	0.018 (2)
C27	0.086 (3)	0.059 (2)	0.133 (4)	−0.023 (2)	0.015 (3)	0.004 (2)
C28	0.069 (2)	0.062 (2)	0.104 (3)	−0.0292 (17)	0.0109 (19)	−0.0153 (19)
C29	0.096 (3)	0.087 (3)	0.161 (5)	−0.045 (3)	0.018 (3)	−0.055 (3)
C30	0.105 (3)	0.128 (4)	0.144 (4)	−0.075 (3)	0.029 (3)	−0.080 (3)
C31	0.073 (2)	0.120 (3)	0.081 (2)	−0.060 (2)	0.0130 (17)	−0.048 (2)
C32	0.087 (2)	0.166 (4)	0.073 (2)	−0.072 (3)	−0.0019 (19)	−0.052 (3)
C33	0.079 (2)	0.180 (5)	0.0632 (19)	−0.063 (3)	−0.0178 (17)	−0.033 (2)
C34	0.0666 (18)	0.111 (3)	0.0562 (16)	−0.0403 (18)	−0.0193 (14)	−0.0155 (16)
C35	0.0541 (15)	0.084 (2)	0.0579 (16)	−0.0384 (15)	0.0067 (12)	−0.0243 (15)
C36	0.0571 (16)	0.0684 (19)	0.0662 (17)	−0.0337 (15)	0.0049 (13)	−0.0154 (14)
N7	0.0637 (13)	0.0574 (14)	0.0516 (12)	−0.0198 (12)	−0.0285 (10)	0.0084 (10)
C37	0.0526 (14)	0.0575 (16)	0.0452 (13)	−0.0186 (12)	−0.0207 (11)	0.0040 (11)
C38	0.0716 (17)	0.0683 (18)	0.0506 (14)	−0.0268 (15)	−0.0311 (13)	0.0003 (13)
C39	0.0719 (17)	0.0559 (17)	0.0599 (15)	−0.0240 (14)	−0.0277 (13)	−0.0074 (13)
C40	0.0526 (14)	0.0529 (15)	0.0550 (14)	−0.0199 (12)	−0.0217 (11)	0.0012 (12)
C43	0.0640 (19)	0.0543 (17)	0.0762 (19)	−0.0180 (15)	−0.0359 (16)	−0.0002 (14)
O1	0.060 (3)	0.084 (4)	0.140 (5)	−0.017 (2)	−0.045 (3)	−0.001 (3)
O2	0.070 (3)	0.063 (4)	0.114 (6)	−0.012 (2)	−0.031 (3)	0.019 (3)
O1B	0.150 (12)	0.061 (7)	0.27 (2)	−0.038 (8)	−0.175 (14)	0.028 (10)
O2B	0.113 (12)	0.055 (6)	0.163 (17)	−0.046 (8)	−0.094 (12)	0.039 (8)
C41	0.0494 (13)	0.0536 (15)	0.0466 (13)	−0.0177 (11)	−0.0212 (10)	0.0014 (11)
C44	0.0609 (16)	0.0626 (18)	0.0563 (16)	−0.0191 (15)	−0.0303 (13)	−0.0017 (14)
O3	0.109 (3)	0.065 (2)	0.069 (3)	−0.017 (2)	−0.059 (2)	0.0042 (19)
O4	0.103 (4)	0.081 (3)	0.108 (5)	0.004 (3)	−0.073 (3)	−0.033 (3)
O3B	0.216 (12)	0.129 (9)	0.073 (6)	−0.120 (9)	−0.090 (7)	0.048 (6)
O4B	0.138 (9)	0.080 (6)	0.077 (6)	−0.067 (7)	−0.066 (6)	0.014 (4)
C42	0.0540 (14)	0.0502 (15)	0.0501 (13)	−0.0155 (12)	−0.0215 (11)	−0.0008 (11)
N12	0.0634 (13)	0.0537 (13)	0.0542 (12)	−0.0239 (11)	−0.0294 (10)	0.0053 (10)
N13	0.0614 (13)	0.0530 (13)	0.0548 (12)	−0.0228 (11)	−0.0295 (10)	0.0027 (10)
O5	0.259 (4)	0.091 (2)	0.181 (3)	−0.015 (2)	−0.168 (3)	−0.038 (2)
O6	0.145 (2)	0.108 (2)	0.0991 (18)	−0.0198 (17)	−0.0903 (18)	−0.0079 (15)
O7	0.0750 (13)	0.0851 (15)	0.0726 (13)	−0.0197 (12)	−0.0455 (11)	0.0188 (11)
O8	0.123 (2)	0.0525 (13)	0.122 (2)	−0.0230 (13)	−0.0799 (17)	0.0138 (13)
O9	0.139 (3)	0.0675 (17)	0.177 (3)	0.0176 (16)	−0.102 (2)	−0.0270 (18)
O10	0.127 (2)	0.095 (2)	0.159 (3)	0.0114 (17)	−0.112 (2)	−0.0156 (18)
O11	0.159 (3)	0.101 (2)	0.163 (3)	−0.0201 (19)	−0.130 (2)	−0.0109 (19)
O12	0.187 (3)	0.0809 (18)	0.116 (2)	−0.0481 (19)	−0.107 (2)	−0.0005 (15)
C45	0.0580 (15)	0.0523 (15)	0.0498 (13)	−0.0201 (12)	−0.0269 (11)	0.0032 (11)
C46	0.097 (2)	0.0518 (17)	0.0747 (19)	−0.0153 (16)	−0.0518 (17)	0.0024 (14)
C47	0.113 (3)	0.0549 (18)	0.089 (2)	−0.0175 (18)	−0.059 (2)	−0.0120 (16)
C48	0.0746 (18)	0.0665 (19)	0.0618 (16)	−0.0175 (15)	−0.0382 (14)	−0.0072 (14)
C49	0.0473 (13)	0.0580 (16)	0.0498 (13)	−0.0184 (12)	−0.0221 (11)	0.0036 (11)
C50	0.0552 (14)	0.0515 (15)	0.0548 (14)	−0.0225 (12)	−0.0235 (11)	0.0014 (11)
C51	0.120 (3)	0.090 (3)	0.097 (3)	−0.016 (2)	−0.072 (2)	−0.018 (2)
C52	0.0511 (15)	0.0621 (18)	0.0705 (17)	−0.0218 (13)	−0.0271 (13)	0.0111 (14)
C53	0.0534 (14)	0.0505 (15)	0.0501 (13)	−0.0245 (12)	−0.0233 (11)	0.0052 (11)
C54	0.0721 (17)	0.0458 (15)	0.0662 (16)	−0.0172 (13)	−0.0413 (14)	0.0062 (12)
C55	0.089 (2)	0.0464 (15)	0.0704 (17)	−0.0236 (15)	−0.0430 (16)	0.0002 (13)
C56	0.0682 (17)	0.0582 (17)	0.0555 (15)	−0.0278 (14)	−0.0328 (13)	0.0067 (12)
C57	0.0543 (14)	0.0538 (16)	0.0586 (15)	−0.0196 (12)	−0.0272 (12)	0.0062 (12)
C58	0.0599 (15)	0.0466 (14)	0.0560 (14)	−0.0190 (12)	−0.0233 (12)	−0.0010 (11)
C59	0.111 (3)	0.074 (2)	0.081 (2)	−0.039 (2)	−0.061 (2)	0.0057 (17)
C60	0.076 (2)	0.068 (2)	0.095 (2)	−0.0058 (17)	−0.0465 (18)	−0.0026 (18)

### Geometric parameters (Å, °)

**Table:**

Cd1—N6	2.327 (2)	C31—C32	1.398 (6)
Cd1—N2	2.343 (2)	C31—C35	1.412 (4)
Cd1—N4	2.350 (2)	C32—C33	1.336 (6)
Cd1—N1	2.350 (2)	C32—H32	0.9300
Cd1—N3	2.355 (2)	C33—C34	1.395 (5)
Cd1—N5	2.367 (2)	C33—H33	0.9300
N1—C1	1.327 (4)	C34—H34	0.9300
N1—C12	1.358 (4)	C35—C36	1.443 (4)
N2—C10	1.321 (4)	N7—N7^i^	1.240 (4)
N2—C11	1.353 (3)	N7—C37	1.430 (3)
N3—C13	1.330 (3)	C37—C38	1.379 (4)
N3—C24	1.357 (3)	C37—C42	1.387 (3)
N4—C22	1.320 (4)	C38—C39	1.368 (4)
N4—C23	1.354 (3)	C38—H38	0.9300
N5—C25	1.331 (4)	C39—C40	1.383 (3)
N5—C36	1.353 (4)	C39—H39	0.9300
N6—C34	1.331 (4)	C40—C41	1.405 (4)
N6—C35	1.342 (4)	C40—C43	1.492 (4)
C1—C2	1.404 (5)	C43—O1B	1.164 (10)
C1—H1	0.9300	C43—O1	1.227 (6)
C2—C3	1.344 (6)	C43—O2	1.266 (7)
C2—H2	0.9300	C43—O2B	1.274 (12)
C3—C4	1.417 (6)	O2—H2A	0.8587
C3—H3	0.9300	O2B—H2A	0.8314
C4—C12	1.409 (4)	C41—C42	1.385 (3)
C4—C5	1.439 (6)	C41—C44	1.500 (3)
C5—C6	1.321 (6)	C44—O3B	1.201 (7)
C5—H5	0.9300	C44—O3	1.209 (5)
C6—C7	1.428 (6)	C44—O4	1.273 (6)
C6—H6	0.9300	C44—O4B	1.283 (9)
C7—C11	1.409 (4)	O4—H4A	0.8427
C7—C8	1.414 (6)	O4B—H4A	0.8941
C8—C9	1.328 (6)	C42—H42	0.9300
C8—H8	0.9300	N12—N13	1.242 (3)
C9—C10	1.388 (4)	N12—C45	1.425 (3)
C9—H9	0.9300	N13—C53	1.435 (3)
C10—H10	0.9300	O5—C51	1.189 (5)
C11—C12	1.432 (4)	O6—C51	1.298 (4)
C13—C14	1.383 (4)	O6—H6A	1.1480
C13—H13	0.9300	O7—C52	1.251 (3)
C14—C15	1.360 (4)	O7—H6A	1.2413
C14—H14	0.9300	O8—C52	1.236 (4)
C15—C16	1.397 (4)	O9—C60	1.203 (4)
C15—H15	0.9300	O10—C60	1.271 (4)
C16—C24	1.402 (4)	O10—H10A	1.1042
C16—C17	1.434 (5)	O11—C59	1.256 (4)
C17—C18	1.320 (5)	O11—H10A	1.4085
C17—H17	0.9300	O12—C59	1.212 (4)
C18—C19	1.435 (5)	C45—C50	1.369 (3)
C18—H18	0.9300	C45—C46	1.375 (4)
C19—C23	1.400 (4)	C46—C47	1.379 (4)
C19—C20	1.417 (5)	C46—H46	0.9300
C20—C21	1.351 (5)	C47—C48	1.390 (4)
C20—H20	0.9300	C47—H47	0.9300
C21—C22	1.381 (4)	C48—C49	1.400 (4)
C21—H21	0.9300	C48—C51	1.536 (4)
C22—H22	0.9300	C49—C50	1.394 (3)
C23—C24	1.449 (4)	C49—C52	1.513 (4)
C25—C26	1.382 (5)	C50—H50	0.9300
C25—H25	0.9300	C53—C54	1.366 (4)
C26—C27	1.340 (6)	C53—C58	1.379 (3)
C26—H26	0.9300	C54—C55	1.375 (4)
C27—C28	1.396 (6)	C54—H54	0.9300
C27—H27	0.9300	C55—C56	1.380 (4)
C28—C36	1.406 (4)	C55—H55	0.9300
C28—C29	1.444 (6)	C56—C57	1.410 (4)
C29—C30	1.318 (7)	C56—C59	1.522 (4)
C29—H29	0.9300	C57—C58	1.395 (3)
C30—C31	1.433 (6)	C57—C60	1.524 (4)
C30—H30	0.9300	C58—H58	0.9300
			
N6—Cd1—N2	94.62 (8)	C28—C29—H29	119.5
N6—Cd1—N4	105.02 (7)	C29—C30—C31	122.7 (4)
N2—Cd1—N4	157.03 (8)	C29—C30—H30	118.7
N6—Cd1—N1	155.72 (9)	C31—C30—H30	118.7
N2—Cd1—N1	71.50 (8)	C32—C31—C35	117.8 (4)
N4—Cd1—N1	93.53 (8)	C32—C31—C30	123.8 (4)
N6—Cd1—N3	93.24 (8)	C35—C31—C30	118.4 (4)
N2—Cd1—N3	96.00 (7)	C33—C32—C31	119.7 (3)
N4—Cd1—N3	71.47 (8)	C33—C32—H32	120.1
N1—Cd1—N3	107.65 (7)	C31—C32—H32	120.1
N6—Cd1—N5	71.49 (9)	C32—C33—C34	119.8 (4)
N2—Cd1—N5	107.14 (7)	C32—C33—H33	120.1
N4—Cd1—N5	90.59 (8)	C34—C33—H33	120.1
N1—Cd1—N5	93.15 (8)	N6—C34—C33	122.3 (4)
N3—Cd1—N5	152.94 (8)	N6—C34—H34	118.8
C1—N1—C12	119.9 (3)	C33—C34—H34	118.8
C1—N1—Cd1	125.3 (2)	N6—C35—C31	121.7 (3)
C12—N1—Cd1	114.47 (18)	N6—C35—C36	119.1 (2)
C10—N2—C11	118.5 (3)	C31—C35—C36	119.2 (3)
C10—N2—Cd1	125.79 (19)	N5—C36—C28	121.5 (3)
C11—N2—Cd1	115.24 (19)	N5—C36—C35	118.5 (3)
C13—N3—C24	118.3 (2)	C28—C36—C35	119.9 (3)
C13—N3—Cd1	126.23 (18)	N7^i^—N7—C37	114.0 (3)
C24—N3—Cd1	115.46 (16)	C38—C37—C42	119.8 (2)
C22—N4—C23	117.9 (2)	C38—C37—N7	116.4 (2)
C22—N4—Cd1	126.4 (2)	C42—C37—N7	123.9 (2)
C23—N4—Cd1	115.55 (16)	C39—C38—C37	120.1 (2)
C25—N5—C36	118.4 (3)	C39—C38—H38	119.9
C25—N5—Cd1	126.9 (2)	C37—C38—H38	119.9
C36—N5—Cd1	114.70 (19)	C38—C39—C40	121.4 (2)
C34—N6—C35	118.7 (3)	C38—C39—H39	119.3
C34—N6—Cd1	125.1 (2)	C40—C39—H39	119.3
C35—N6—Cd1	116.16 (18)	C39—C40—C41	118.7 (2)
N1—C1—C2	121.8 (4)	C39—C40—C43	117.5 (2)
N1—C1—H1	119.1	C41—C40—C43	123.7 (2)
C2—C1—H1	119.1	O1B—C43—O1	34.1 (9)
C3—C2—C1	119.0 (4)	O1B—C43—O2	115.9 (11)
C3—C2—H2	120.5	O1—C43—O2	123.8 (7)
C1—C2—H2	120.5	O1B—C43—O2B	125.6 (12)
C2—C3—C4	121.1 (3)	O1—C43—O2B	116.8 (11)
C2—C3—H3	119.4	O2—C43—O2B	24.9 (12)
C4—C3—H3	119.4	O1B—C43—C40	122.9 (9)
C12—C4—C3	116.6 (4)	O1—C43—C40	122.5 (5)
C12—C4—C5	118.4 (4)	O2—C43—C40	113.2 (6)
C3—C4—C5	125.0 (4)	O2B—C43—C40	111.2 (9)
C6—C5—C4	122.0 (4)	C43—O2—H2A	108.0
C6—C5—H5	119.0	C43—O2B—H2A	109.2
C4—C5—H5	119.0	C42—C41—C40	119.6 (2)
C5—C6—C7	121.5 (4)	C42—C41—C44	118.6 (2)
C5—C6—H6	119.2	C40—C41—C44	121.8 (2)
C7—C6—H6	119.2	O3B—C44—O3	48.8 (6)
C11—C7—C8	117.8 (3)	O3B—C44—O4	101.9 (7)
C11—C7—C6	118.5 (4)	O3—C44—O4	124.8 (4)
C8—C7—C6	123.7 (4)	O3B—C44—O4B	121.9 (7)
C9—C8—C7	119.7 (3)	O3—C44—O4B	113.5 (6)
C9—C8—H8	120.1	O4—C44—O4B	35.3 (5)
C7—C8—H8	120.1	O3B—C44—C41	123.7 (5)
C8—C9—C10	119.4 (4)	O3—C44—C41	122.6 (3)
C8—C9—H9	120.3	O4—C44—C41	112.5 (4)
C10—C9—H9	120.3	O4B—C44—C41	111.6 (5)
N2—C10—C9	123.4 (3)	C44—O4—H4A	112.4
N2—C10—H10	118.3	C44—O4B—H4A	107.8
C9—C10—H10	118.3	C41—C42—C37	120.3 (2)
N2—C11—C7	121.1 (3)	C41—C42—H42	119.8
N2—C11—C12	118.5 (2)	C37—C42—H42	119.8
C7—C11—C12	120.4 (3)	N13—N12—C45	115.8 (2)
N1—C12—C4	121.5 (3)	N12—N13—C53	112.3 (2)
N1—C12—C11	119.3 (2)	C51—O6—H6A	114.1
C4—C12—C11	119.2 (3)	C52—O7—H6A	113.3
N3—C13—C14	123.3 (3)	C60—O10—H10A	104.8
N3—C13—H13	118.3	C59—O11—H10A	104.9
C14—C13—H13	118.3	C50—C45—C46	119.4 (2)
C15—C14—C13	118.7 (3)	C50—C45—N12	114.6 (2)
C15—C14—H14	120.6	C46—C45—N12	125.9 (2)
C13—C14—H14	120.6	C45—C46—C47	118.4 (3)
C14—C15—C16	120.1 (3)	C45—C46—H46	120.8
C14—C15—H15	120.0	C47—C46—H46	120.8
C16—C15—H15	120.0	C46—C47—C48	123.0 (3)
C15—C16—C24	117.9 (3)	C46—C47—H47	118.5
C15—C16—C17	123.2 (3)	C48—C47—H47	118.5
C24—C16—C17	118.9 (3)	C47—C48—C49	118.5 (2)
C18—C17—C16	121.0 (3)	C47—C48—C51	112.5 (3)
C18—C17—H17	119.5	C49—C48—C51	128.9 (3)
C16—C17—H17	119.5	C50—C49—C48	117.3 (2)
C17—C18—C19	122.4 (3)	C50—C49—C52	114.5 (2)
C17—C18—H18	118.8	C48—C49—C52	128.1 (2)
C19—C18—H18	118.8	C45—C50—C49	123.3 (2)
C23—C19—C20	117.2 (3)	C45—C50—H50	118.4
C23—C19—C18	118.6 (3)	C49—C50—H50	118.4
C20—C19—C18	124.1 (3)	O5—C51—O6	121.7 (3)
C21—C20—C19	119.8 (3)	O5—C51—C48	119.4 (3)
C21—C20—H20	120.1	O6—C51—C48	118.8 (3)
C19—C20—H20	120.1	O8—C52—O7	122.8 (3)
C20—C21—C22	118.6 (3)	O8—C52—C49	115.9 (2)
C20—C21—H21	120.7	O7—C52—C49	121.3 (3)
C22—C21—H21	120.7	C54—C53—C58	119.8 (2)
N4—C22—C21	124.2 (3)	C54—C53—N13	124.1 (2)
N4—C22—H22	117.9	C58—C53—N13	116.1 (2)
C21—C22—H22	117.9	C53—C54—C55	118.8 (2)
N4—C23—C19	122.2 (3)	C53—C54—H54	120.6
N4—C23—C24	118.7 (2)	C55—C54—H54	120.6
C19—C23—C24	119.1 (3)	C54—C55—C56	123.2 (3)
N3—C24—C16	121.7 (2)	C54—C55—H55	118.4
N3—C24—C23	118.5 (2)	C56—C55—H55	118.4
C16—C24—C23	119.8 (2)	C55—C56—C57	118.1 (2)
N5—C25—C26	123.1 (4)	C55—C56—C59	114.0 (3)
N5—C25—H25	118.5	C57—C56—C59	127.8 (2)
C26—C25—H25	118.5	C58—C57—C56	117.9 (2)
C27—C26—C25	119.0 (4)	C58—C57—C60	113.7 (2)
C27—C26—H26	120.5	C56—C57—C60	128.5 (2)
C25—C26—H26	120.5	C53—C58—C57	122.2 (2)
C26—C27—C28	120.6 (4)	C53—C58—H58	118.9
C26—C27—H27	119.7	C57—C58—H58	118.9
C28—C27—H27	119.7	O12—C59—O11	122.1 (3)
C27—C28—C36	117.4 (4)	O12—C59—C56	117.3 (3)
C27—C28—C29	123.8 (4)	O11—C59—C56	120.6 (3)
C36—C28—C29	118.7 (4)	O9—C60—O10	120.9 (3)
C30—C29—C28	120.9 (4)	O9—C60—C57	119.1 (3)
C30—C29—H29	119.5	O10—C60—C57	119.9 (3)

Symmetry codes: (i) −*x*+1, −*y*, −*z*+1.

### Hydrogen-bond geometry (Å, °)

**Table:**

*D*—H···*A*	*D*—H	H···*A*	*D*···*A*	*D*—H···*A*
O6—H6A···O7	1.15	1.24	2.386 (4)	174.
O10—H10A···O11	1.10	1.41	2.367 (4)	141.
